# 0731. Effects of sildenafil in a porcine model of endotoxemia

**DOI:** 10.1186/2197-425X-2-S1-P53

**Published:** 2014-09-26

**Authors:** DA Kemper, DA Otsuki, DR Maia, N Queiroz-Hazarbassanov, CO Massoco, JOC Auler, DT Fantoni

**Affiliations:** Laboratory of Anesthesiology (LIM08), Faculdade de Medicina da Universidade de São Paulo, São Paulo, Brazil; Surgery, Faculdade de Medicina Veterinária e Zootecnia da Universidade de São Paulo, São Paulo, Brazil; Pathology, Faculdade de Medicina Veterinária e Zootecnia da Universidade de São Paulo, São Paulo, Brazil

## Introduction

Sepsis-induced lung injury is one of the major causes of morbidity and mortality in intensive care patients [1]. The clinical manifestations include pulmonary hypertension, formation of extravascular lung water (EVLW), and deterioration of pulmonary gas exchange. Administration of sildenafil, a selective inhibitor of isoenzyme phosphodiesterase-5, in patients with pulmonary hypertension improves oxygenation and ameliorates pulmonary hypertension [2].

## Objectives

To evaluate the effect of sildenafil on endotoxin-induced lung injury in pigs.

## Methods

Twenty anesthetized and mechanically ventilated pigs were randomized after baseline (BL) measurements to Control (saline solution) or Sildenafil (100mg) group. After 30 minutes of saline/sildenafil administration, all animals were submitted to a continuous lipopolysaccharide (LPS) infusion (4mcg/kg/min) until the end of study. Hemodynamics and oxygenation parameters were evaluated at BL, 30, 60, 120 and 180 minutes after LPS (LPS60, LPS120 and LPS180). Plasma cytokines (TNF-alpha, IL-1beta, IL-6 and IL-10) were evaluated at BL and LPS180. The parametric data were analyzed using ANOVA for repeated measurements and nonparametric data with Kruskall-Wallis and the Mann-Whitney U test.

## Results

**Figure 1:**
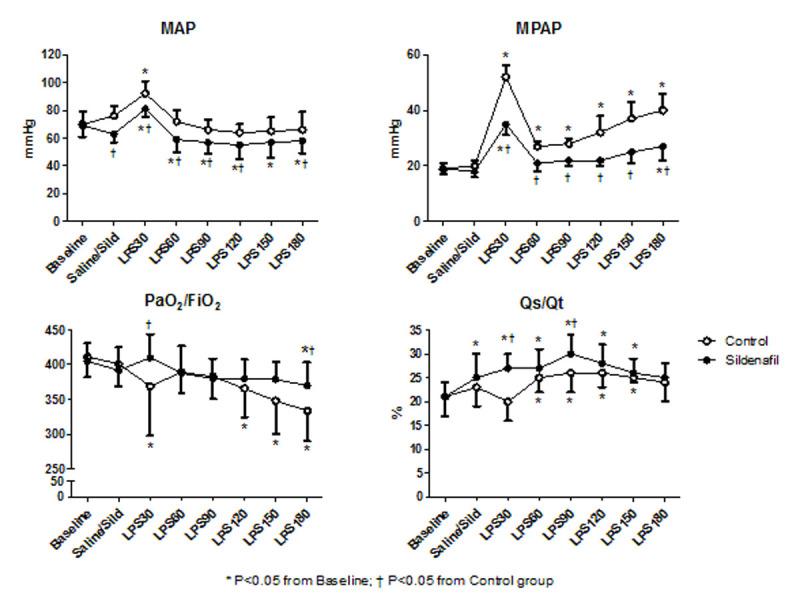
Hemodynamics and oxygenation

Endotoxemia induced a significant pulmonary hypertension with more than a twofold increase in mean arterial pulmonary pressure and pulmonary vascular resistance index, and also a decrease in PaO_2_/FiO_2_. Mean arterial pulmonary and mean arterial pressures were significantly lower in Sildenafil group. Sildenafil improved arterial oxygen tension but also increased the shunt fraction (Figure1).

All cytokines increased after LPS infusion in both groups and no difference was observed between the animals receiving sildenafil and normal saline.

## Conclusions

Sildenafil administration improved pulmonary hypertension and oxygenation in LPS-induced lung injury but increased shunt fraction and promoted systemic hypotension. It remains unclear whether sildenafil may be beneficial in sepsis patients.
